# Comparison of Bone Metastases between ^18^F-NaF PET/CT, ^18^F-NaF PET, and Planar ^99m^Tc-MDP Bone Scintigraphy in Patients with Newly Diagnosed Nasopharyngeal Carcinoma

**DOI:** 10.1155/2022/5975338

**Published:** 2022-04-13

**Authors:** Dong Wang, YiYang Yang, ZhenPei Zeng, Jing Ye, ChengMao Guo, ShiSang Huang, XuFeng Guo, JingXing Xiao

**Affiliations:** ^1^Department of Nuclear Medicine (PET-CT Center), Affiliated Hospital of Guangdong Medical University, Zhanjiang, China; ^2^Hospice Unit, Affiliated Hospital of Guangdong Medical University, Zhanjiang, China; ^3^Department of Ophthalmology, The Second Affiliated Hospital of Guangdong Medicinal University, Zhanjiang, China

## Abstract

**Purpose:**

Our study aims to compare the diagnostic value of ^18^F-NaF positron emission tomography-computed tomography (PET/CT), ^18^F-NaF PET, and planar ^99m^Tc-MDP bone scintigraphy for detection of bone metastases in patients with newly diagnosed nasopharyngeal carcinoma (NPC).

**Methods:**

Our study retrospectively analyzed 58 patients with pathologically proven NPC. They all underwent both ^18^F-NaF PET/CT and planar ^99m^Tc-MDP bone scintigraphy within a 7-day interval. Bone metastases were confirmed by follow-up using PET/CT, contrast-enhanced computed tomography (CT), and magnetic resonance imaging (MRI). These three examinations were compared using per-patient-based analysis and per-lesion-based analysis.

**Results:**

19 patients (32.7%) were classified as having bone metastatic disease in their final diagnosis. The patient-based diagnostic performances (sensitivity, specificity, and overall accuracy) were as follows: ^18^F-NaF PET/CT (100%, 92.3%, and 94.8%), ^18^F-NaF PET (100%, 53.8%, and 69.0%), and planar ^99m^Tc-MDP bone scintigraphy (78.9%, 74.4%, and 75.9%). The overall accuracy of ^18^F-NaF PET/CT was significantly more favorable compared to ^18^F-NaF PET (*p*=0.002) and to planar ^99m^Tc-MDP bone scintigraphy (*p*=0.044). The lesion-based diagnostic performances (sensitivity, specificity, and overall accuracy) were as follows: ^18^F-NaF PET/CT (98.5%, 93.9%, and 96.6%), ^18^F-NaF PET (98.5%, 57.1%, and 81.1%), and planar ^99m^Tc-MDP bone scintigraphy (69.9%, 85.7%, and 76.4%).

**Conclusion:**

^18^F-NaF PET/CT outperforms ^18^F-NaF PET or planar ^99m^Tc-MDP bone scintigraphy in detecting bone metastases with newly diagnosed NPC on a patient-based and lesion-based analysis.

## 1. Introduction

Nasopharyngeal carcinoma (NPC) is an uncommon cancer worldwide; >70% of new cases are in East and Southeast Asia [1]. Local advanced NPC has a tendency to bone metastases. Autopsy studies show that distant metastases are as frequent as 38–87% and that bone metastases occur in 70–80% of patients with distant metastases [2, 3]. The actual frequency of such metastases may be greater than the reported data owing to the low autopsy rate in Asia. Metastatic bone disease is the most frequent malignancy of the skeletal system [4]. The metastatic bone disease may cause serious endocrinologic, hematologic, neurologic, and orthopedic complications and intolerable pain [5]. Early detection of bone metastases and accurate NPC staging is important for improving both patient quality of life and therapeutic effects.

Prior to treating NPC, the presence of bone metastases should be evaluated. The management of patients with bone metastases is quite different. If bone metastases are diagnosed, the clinical stage is upgraded to M1, which has implications for changing therapeutic strategies, such as changing from a radiotherapy-based treatment regimen to a chemotherapy-based regimen [1]. An accurate detection of the presence of bone metastases is important throughout the disease course of NPC to select an optimal treatment strategy and to reduce potential complications.

^99m^Tc-methylene diphosphonate (MDP) bone scintigraphy is widely used as a noninvasive conventional modality for detecting bone metastases, especially in developing countries. However, this method cannot obtain cross-sectional images of all the lesions, and they have lower resolution than other imaging techniques when comparing with other advanced imaging methods, such as MRI or PET/CT. In early bone metastasis, especially when the majority of tumor cells are confined in the bone marrow, the diagnostic efficiency of planar ^99m^Tc-MDP bone scintigraphy is far from satisfactory [6].

As a molecular imaging technology, PET/CT can indicate the degree of metabolic function of malignancy and the clinical stage, response to therapy, and tumor recurrence, whereas conventional imaging modalities can only reveal morphological and anatomical information [7, 8] ^18^F-NaF was approved by the U.S. Food and Drug Administration as a bone-seeking diagnostic molecular imaging agent in 1972 [9]. Because ^18^F-NaF has better pharmacokinetic characteristics than ^99m^Tc-MDP, ^18^F-NaF regained clinical attention with the development of PET/CT.

Some reports have compared the diagnostic value of ^18^F-NaF PET/CT with that of planar ^99m^Tc-MDP bone scintigraphy for detecting bone metastases of lung and prostate cancer [10–12]. As shown in our preliminary study, the results of the diagnostic performance of ^18^F-NaF PET/CT are promising [13]. However, to the best of our knowledge, no study has compared the clinical value of ^18^F-NaF PET/CT with that of planar ^99m^Tc-MDP bone scintigraphy for the detection of bone metastases in newly diagnosed nasopharyngeal carcinoma. Thus, the aim was to perform a diagnostic accuracy study on the detection of bone metastases by means of ^18^F-NaF PET/CT in comparison with ^18^F-NaF PET and planar ^99m^Tc-MDP bone scintigraphy in newly diagnosed patients with NPC.

## 2. Materials and Methods

### 2.1. Patients

We reviewed the medical records of patients with pathologically proven NPC from July 2017 to June 2020 who underwent both ^18^F-NaF PET/CT and planar ^99m^Tc-MDP bone scintigraphy within an interval of 7 days. Exclusion criteria were patients receiving chemotherapy or radiotherapy treatment, prior radiotherapy of bone metastases, prior malignancy, bone metabolism disorder, osteomyelitis, and any conditions contraindicated for MRI scan or a CT contrast agent. We obtained informed consent from patients before both examinations. Our retrospective review of imaging studies was approved by the institutional review board.

### 2.2. ^18^F-NaF PET/CT Protocols

^18^F-NaF PET/CT was conducted in accordance with the guidelines from the Society of Nuclear Medicine, and the European Association of Nuclear Medicine. ^18^F-NaF was produced by using a cyclotron (HM-10, Japan) and an automatic synthesis module (Beijing PET Technology Co., Ltd., Beijing, China) in our centre. The radiochemical purity of ^18^F-NaF was greater than 95%. The dose of ^18^F-NaF was 3.75 MBq/kg. Approximately 1 hour after the injection, the examination began with a PET/CT 690 scanner (General Electric Medical Systems, Milwaukee, Wisconsin, USA). For ^18^F-NaF PET/CT, the scanned area ranged from the feet to the cranium. The emission image acquisition time was 120 seconds per bed position. PET image data were reconstructed by applying attenuation correction based on the CT data using the ordered subset expectation examination algorithm.

#### 2.2.1. Planar ^99m^Tc-MDP Bone Scintigraphy Protocols

Planar ^99m^Tc-MDP bone scintigraphy was obtained approximately 2–4 h after the intravenous injection of 925 MBq (25 mCi) of ^99m^Tc-MDP in the anterior and posterior projections using dual-head gamma camera (Symbia E, Siemens) equipped with a low-energy, high-resolution collimator at a scan speed of 20 cm/min. The photopeak was centred at 140 keV with a 20% window. SPECT or SPECT/CT images were not acquired.

### 2.3. Image Interpretation

Two experienced nuclear medicine physicians independently evaluated the ^18^F-NaF PET/CT, ^18^F-NaF PET, and planar ^99m^Tc-MDP bone scintigraphy images in a random order for each patient. They were blinded to other imaging results and the final results of the lesions. The PET/CT, PET, and planar ^99m^Tc-MDP bone scintigraphy images were interpreted at different times and in a different order so that the interpretation would not be influenced. Discrepancies between the two readers were found in two cases in our study, one in which two experts found differences in the number of bone lesions in the three examination of PET/CT, PET, or bone scintigraphy, and the other in which two experts judged the nature of the bone lesions in the three examination means. The solution was to include the opinion of a third expert with more than 10 years of nuclear medicine certification if there was a discrepancy, and ultimately to judge the nature of the lesion by the vote of the three experts.

### 2.4. Definition of Bone Metastases

For the interpretation of the scans, visual analysis was used instead of semiquantitative analysis (i.e., SUV cutoffs). For the ^18^F-NaF PET/CT scans, areas of focally increased ^18^F-NaF uptake were recorded as malignant unless a benign etiology (e.g., degenerative changes or hemangioma) for this uptake was identified at the same location on the corresponding CT images. The CT component of PET/CT was used to determine whether bone lesions identified on PET had an osteoblastic or osteolytic appearance. Bone destruction or osteoblastic manifestation of the bone (local and asymmetric lesions with increased density) was targeted as malignancy. The final bone metastasis of a given site was determined based on either pathological examination from CT-guided or surgical biopsies or the results of follow-up by MRI, contrast-enhanced CT, or PET/CT for more than six months for every patient. The suspicious lesions detected by PET/CT or ^99m^Tc-MDP bone scintigraphy were confirmed to be metastasis when the tissues were pathologically proved to be metastatic, or the lesions became larger during the follow-up periods or decreased in size after treatment. On the contrary, they were diagnosed as nonmetastatic lesions when no change in size was observed during follow-up examinations. The final diagnosis was arrived at by consensus at a conference held by the multidisciplinary group of NPC in our hospital.

#### 2.4.1. Patient-Based Analysis

If more than one lesion was present in the same patient with a discordant diagnostic classification, the following rules were used: a patient who had at least one true positive lesion was classified as true positive; in the absence of a true-positive lesion, a false-negative lesion superseded a true-negative or a false-positive lesion.

#### 2.4.2. Lesion-Based Analysis

The lesion-based analysis involves the skeletal system, excluding the rib, pelvic bone, cervical vertebra, lumbar vertebra, thoracic vertebra, limb bone, scapula, skull, and the other bone.

### 2.5. Statistical Analysis

The data analyses were performed using the R software (version 3.4.3; http://www.r-project.org). Measures of the diagnostic performances of the ^18^F-NaF PET/CT, ^18^F-NaF PET, and planar ^99m^Tc-MDP bone scintigraphy were calculated from patient-based dichotomous outcomes (0 or ≥1 bone metastasis). A Cochran's Q test was performed to compare the diagnostic performances (sensitivity, specificity, and overall accuracy) of the three imaging methods, and a McNemar test was performed for pairwise comparisons. A *p* value less than 0.05 was considered statistically significant. *p* values for pairwise comparisons are shown unadjusted, but information on adjustments using the Bonferroni–Holm method is presented.

## 3. Results

### 3.1. Final Study Population

Fifty-eight patients (aged 28–67 years) constituted the final study population. Nineteen out of the 58 patients (32.7%) were classified as having metastatic bone disease as their final diagnosis. The most common sites of bone metastases were the thoracic vertebra (*n* = 28), followed by ribs (*n* = 26). Bone metastases were mainly osteolytic lesions (49.6%) (Table 1).

### 3.2. Patient-Based Diagnostic Accuracy Measurements

^18^F-NaF PET/CT misclassified three patients (false positive *n* = 3 and false negative *n* = 0), ^18^F-NaF PET misclassified 18 patients (false positive *n* = 18 and false negative *n* = 0), and planar ^99m^Tc-MDP bone scintigraphy misclassified 14 patients (false positive *n* = 10 and false negative *n* = 4) (Table 2).

**Figure 1:**
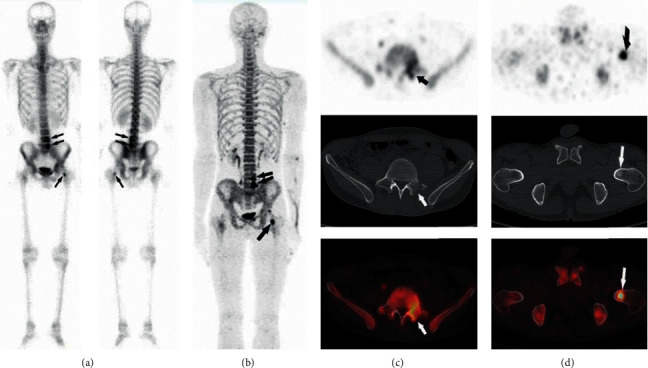
A 36-year-old man with NPC and confirmed as bone metastases by follow-up in the 5th lumbar spine, sacral spine, and left femoral neck. All modalities show clear evidence of metastasis: focal accumulation in planar ^99m^Tc-MDP bone scintigraphy (a) (arrowheads), tracer uptake in ^18^F-NaF PET (b) (arrows), and cortical destruction (c) and osteolysis in CT (d).

**Figure 2:**
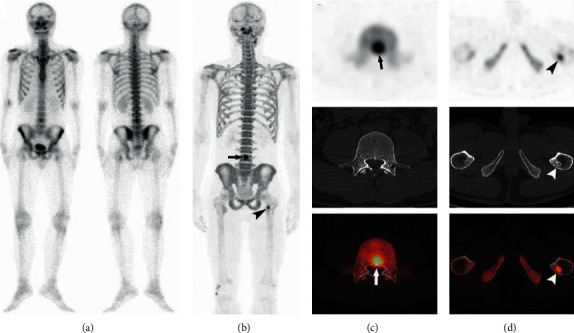
A 28-year-old man with NPC and a single histologically confirmed bone metastasis in the 4th lumbar spine. Planar ^99m^Tc-MDP bone scintigraphy yielded false-negative results (a). Clear identification of metastasis in PET (b) (arrow) and in ^18^F-NaF PET/CT (c) (arrows). In addition, PET identified a lesion on the left upper femur with tracer uptake and considered a bone metastasis (c) (arrows), while CT showed a sclerotic border around the lesion (d) (arrowheads) and suggestive of a benign lesion in PET/CT (correctly according to the reference standard).

**Figure 3:**
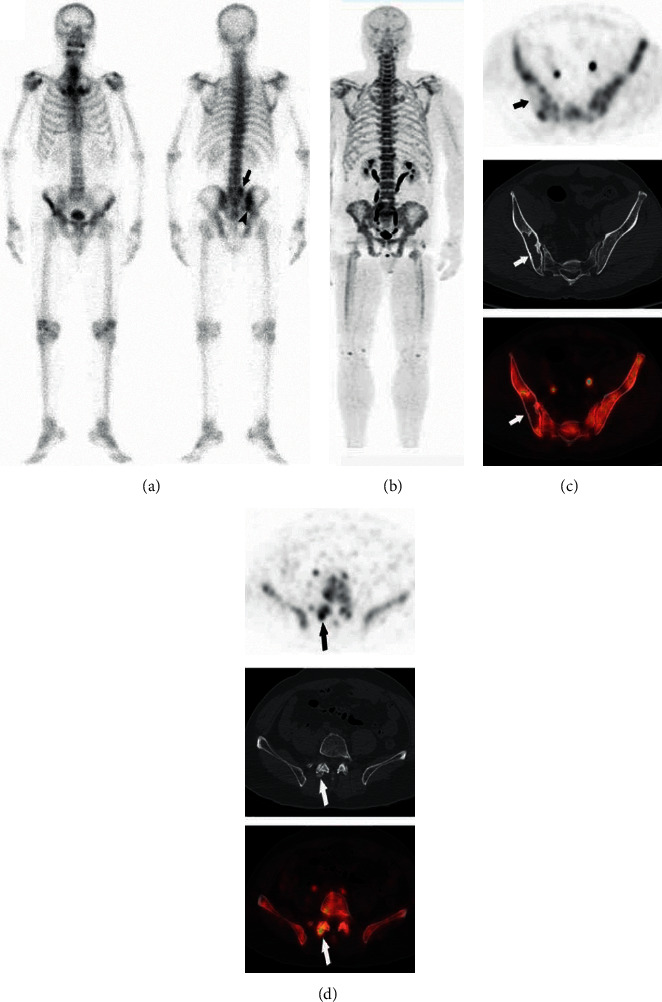
A 59-year-old man with NPC and planar ^99m^Tc-MDP bone scintigraphy with two increased tracer uptake lesions in the 5th lumber spine (arrow) and right iliac (arrowhead) suggestive of bone metastases (a). ^18^F-NaF showed false positives in the lumbar spine and true negatives in the right iliac bone (b). Clear identification of benign lesion of the right iliac bone in ^18^F-NaF PET/CT (b, c) (short arrows). Correlation of PET findings with morphologic changes on CT in the small joint of the right 5th lumbar vertebra (d) (long arrows), suggestive of degenerative changes (correctly according to the reference standard).

In all the nineteen patients with bone metastases, fifteen patients were correctly detected both in planar ^99m^Tc-MDP bone scintigraphy and ^18^F-NaF PET/CT (Figure 1), while four other patients were only correctly detected in ^18^F-NaF PET/CT but were missed in planar ^99m^Tc-MDP bone scintigraphy (Figure 2). Three patients showed false positives in planar ^99m^Tc-MDP bone scintigraphy, ^18^F-NaF PET, and ^18^F-NaF PET/CT. Five patients were misclassified as false positive on planar ^99m^Tc-MDP bone scintigraphy and ^18^F-NaF PET while true negative on ^18^F-NaF PET/CT (Figure 3).

Therefore, on a patient-based level the diagnostic performances (sensitivity, specificity, and overall accuracy) were as follows: ^18^F-NaF PET/CT (100%, 92.3%, and 94.8%), ^18^F-NaF PET (100%, 53.8%, and 69.0%) and planar ^99m^Tc-MDP bone scintigraphy (78.9%, 74.4%, and 75.9%) (Table 2). Pairwise comparisons revealed that the overall accuracy of ^18^F-NaF PET/CT was significantly more favorable compared to ^18^F-NaF PET (*p*=0.002). In addition, a tendency towards a more favorable specificity of ^18^F-NaF PET/CT compared to planar ^99m^Tc-MDP bone scintigraphy was shown (*p*=0.044). No significant differences in the diagnostic performances were found between ^18^F-NaF PET and planar ^99m^Tc-MDP bone scintigraphy (*p*=1.000).

### 3.3. Lesion-Based Diagnostic Accuracy Measurements

In the study, five patients had more than ten bone metastatic lesions, whereas, in the remaining 14 patients, less than ten lesions were present. Using ^18^F-NaF PET/CT, 133 (98.5%) lesions were identified as bone metastases in 19 patients (mean, 7). In contrast, using planar ^99m^Tc-MDP bone scintigraphy, physicians diagnosed 94 lesions as bone metastases (mean, 4.5). The locations of these lesions are described in Table 1.

**Figure 4:**
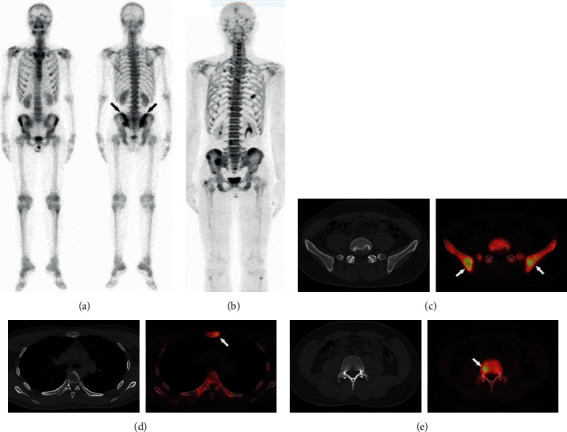
A 31-year-old man with NPC. Planar ^99m^Tc-MDP bone scintigraphy shows bone metastases in both of the iliac bones (a). ^18^F-NaF PET is able to detect more metastatic lesions with significantly better resolution than a conventional bone scan (b). No signs of malignancy were seen in CT (c, d, e). After chemotherapy, follow-up ^18^F-NaF PET/CT suggested a disappearance of the lesions, which was confirmed as a true positive ^18^F-NaF PET/CT prior to treatment.

The lesion-based diagnostic performances (sensitivity, specificity, and overall accuracy) were as follows: ^18^F-NaF PET/CT (98.5%, 93.9%, and 96.6%), ^18^F-NaF PET (98.5%, 57.1%, and 81.1%), and planar ^99m^Tc-MDP bone scintigraphy (78.9%, 74.4%, and 75.9%) (Table 3). Based on the lesion level, the overall accuracy of ^18^F-NaF PET/CT was significantly more favorable compared to ^18^F-NaF PET (*p* < 0.001), and to planar ^99m^Tc-MDP bone scintigraphy (*p*=0.001). (Figure 4).

## 4. Discussion

This study aimed to investigate diagnostic imaging of bone metastases in patients with NPC. To the best of our knowledge, this is the first diagnostic accuracy study to show a significantly more favorable overall accuracy of ^18^F-NaF PET/CT compared to ^18^F-NaF PET and planar ^99m^Tc-MDP bone scintigraphy with NPC.

Several studies have focused on determining the detection accuracy of ^18^F-NaF PET/CT for bone metastases in various cancers [14–17]. However, surprisingly few previous studies have aimed to investigate the diagnostic accuracy of NaF PET/CT or PET of bone metastases with NPC patients. Zhang et al. retrospectively analyzed 45 patients with pathologically proven NPC. The sensitivity, specificity, and agreement rate of ^18^F-NaF PET/CT for detecting metastatic bone lesions were 98.3%, 65.7%, and 92.9%, respectively, and concluded that ^18^F-NaF PET/CT outperforms ^18^F-FDG PET/CT [18]. Also, the sensitivity and accuracy of ^18^F-NaF PET/CT in this study are in line with the previous study [18].

Although only a few studies have compared ^18^F-NaF PET/CT or PET with planar ^99m^Tc-MDP bone scintigraphy for evaluation of bone disease, ^18^F-NaF PET/CT seems more sensitive than conventional bone scanning, showing a higher contrast between normal and abnormal tissue and with the potential for the assessment of small bony structures [19–23]. In this study, the diagnostic value of ^18^F-NaF PET/CT for detection of bone metastases in NPC patients was retrospectively assessed and compared with that of planar ^99m^Tc-MDP bone scintigraphy. ^18^F-NaF PET/CT detected 133 bone metastases (98.5%), whereas planar ^99m^Tc-MDP bone scintigraphy detected only 94 bone metastases (69.6%). These findings are in line with previous studies by Ota et al. [24] on the efficacy of ^18^F-NaF PET/CT and planar ^99m^Tc-MDP bone scintigraphy in the detection of bone metastases with thyroid cancer. Compatibly with the literature data, we observed ^18^F-NaF uptake in both lytic and blastic metastases. Small lesions, which could not be detected by ^99m^Tc-MDP whole-body bone scintigraphy, were easily visualized by the high-resolution power of PET/CT in ^18^F-NaF PET/CT studies. From our results, several important conclusions can be drawn: First, ^18^F-NaF PET/CT performed better than planar ^99m^Tc-MDP bone scintigraphy in determining the metastatic status of patients (almost presence of bone metastases), showing favorable sensitivity and specificity. Second, ^18^F-NaF PET/CT exhibited a significantly higher diagnostic accuracy for the assessment of involved bone regions. The reasons can be explained as follows: firstly, higher uptake of ^18^F-NaF than ^99m^Tc-MDP in the skeleton and faster blood clearance yield a better target/background ratio in a shorter time period. Secondly, bone metastases of nasopharyngeal carcinoma in our case were mainly osteolytic lesions (49.6%) and no obvious morphological changes (35.5%), while there were only a few osteoblastic lesions. ^18^F-NaF uptake in both lytic and blastic metastasis, sectional imaging advantage of the whole body and easy detection of small lesions with improved resolution of PET technology, and better visualization of bone marrow lesions are all contributing factors to the success of ^18^F-NaF PET/CT [9, 25–27] This is important in the way that ^18^F-NaF PET/CT may diagnose metastatic lesions while planar ^99m^Tc-MDP bone scintigraphy is found normal.

Interestingly, although the sensitivity of ^18^F-NaF PET to detect bone metastases from nasopharyngeal carcinoma was significantly better than planar ^99m^Tc-MDP bone scintigraphy, the accuracy was not significantly different from planar ^99m^Tc-MDP bone scintigraphy, mainly because PET showed many false positives in detection. CT plays an important value in helping PET/CT to rule out false positives. ^18^F-NaF PET/CT scan can provide precise information regarding both the morphologic and bone metabolism changes occurring in bone metastases, so the specificity of ^18^F-NaF PET in bone detection can be improved by the use of a PET-CT system. In this study, ^18^F-NaF PET detected (175/233) bone lesions, and all of them were considered to be bone metastases. In contrast, the combination of CT observation revealed that (25/233) of the bone lesions were considered benign because they were at the bone or vertebral margins, and (11/233) of the lesions were considered benign because of the presence of bone lesions with benign features such as sclerotic margins around the lesions. Compared with ^99m^Tc-MDP bone scintigraphy, ^18^F-NaF PET and ^18^F-NaF PET/CT have higher sensitivity. ^18^F-NaF PET had the highest number of false positives on both the patient-based level (18 patients) and lesion-based level (46 lesions), whereas Na PET/CT had the lowest number of false positives on both the patient-based level (3 patients) and lesion-based level (6 lesions). Thus, CT played an important value in excluding false-positive lesions.

However, our study had several limitations. The first was the retrospective nature of the study and the relatively small number of patients with heterogeneity, which might have led to the selection bias. Second, as all the patients had malignant tumors, it was impossible for us to obtain biopsy material in most patients when detecting lesions, which might cause some errors in the final diagnosis. For the two reasons, we suggest that further clinical trials should be undertaken, especially a prospective and multicentre study.

## 5. Conclusion

Our retrospective study with a limited number of patients demonstrated that there were still considerable metastatic lesions that could be detected by ^18^F PET/CT imaging while negative in planar ^99m^Tc-MDP bone scintigraphy. ^18^F-NaF PET/CT is significantly better than ^18^F-NaF PET and planar ^99m^Tc-MDP bone scintigraphy for the detection of bone metastases in patients with newly diagnosed NPC. Although diagnostic superiority could be shown, effects on patient outcomes have to be evaluated in further prospective studies.

## Figures and Tables

**Table 1:** Clinical characteristics and lesion distribution of patients.

Characteristic	Number of patients (%)
No. of patients	58
Age (year), mean (range)	50.4 (28–67)
Stage	
** **I	0
** **II	3 (5.2)
** **III	20 (34.5)
** **IVa	14 (24.1)
** **IVb	21 (36.2)
Site of bone metastases	
** **Ribs	26 (19.3)
** **Pelvic bone	24 (17.8)
** **Cervical vertebra	14 (10.4)
** **Lumbar vertebra	24 (17.8)
** **Thoracic vertebra	28 (20.7)
** **Limb bone	10 (7.4)
** **Scapula	5 (3.7)
** **Skull	2 (1.5)
** **Others	2 (1.5)
Morphologic characteristics of bone metastases	
** **Osteolytic lesions	67 (49.6)
** **Osteoblastic lesions	16 (11.8)
** **No change on CT	52 (35.5)

One patient may have more than one type of lesions.

**Table 2:** Patient-based analysis of lesion on ^18^F-NaF PET/CT, ^18^F-NaF PET, and planar ^99m^Tc-MDP bone scintigraphy (*n* = 58).

	TP	FP	TN	FN	PPV (95% CI)	NPV (95% CI)	Sensitivity (95% CI)	Specificity (95% CI)	Accuracy (95% CI)
PET/CT	19	3	36	0	0.864 (0.760, 1.000)	1.000 (1.000, 1.000)	1.000 (1.000, 1.000)	0.923 (0.821, 1.000)	0.948 (0.879, 1.000)
PET	19	18	21	0	0.514 (0.442, 0.613)	1.000 (1.000, 1.000)	1.000 (1.000, 1.000)	0.538 (0.385, 0.692)	0.690 (0.586, 0.793)
SPECT	15	10	29	4	0.600 (0.469, 0.762)	0.879 (0.784, 0.969)	0.789 (0.579, 0.947)	0.744 (0.590, 0.872)	0.759 (0.655, 0.862)

TP: true positive; FP: false positive; TN: true negative; FN: false negative; NPV: negative predictive value; PPV: positive predictive value.

**Table 3:** Lesion-based analysis of lesion on ^18^F-NaF PET/CT, ^18^F-NaF PET, and planar ^99m^Tc-MDP bone scintigraphy (*n* = 233).

	TP	FP	TN	FN	PPV (95% CI)	NPV (95% CI)	Sensitivity (95% CI)	Specificity (95% CI)	Accuracy (95% CI)
PET/CT	133	6	92	2	0.957 (0.924, 0.985)	0.979 (0.948, 1.000)	0.985 (0.963, 1.000)	0.939 (0.888, 0.980)	0.966 (0.940, 0.987)
PET	133	42	56	2	0.760 (0.720, 0.804)	0.966 (0.914, 1.000)	0.985 (0.963, 1.000)	0.571 (0.469, 0.663)	0.811 (0.768, 0.854)
SPECT	94	14	84	41	0.870 (0.816, 0.927)	0.672 (0.615, 0.733)	0.696 (0.615, 0.770)	0.857 (0.786, 0.918)	0.764 (0.712, 0.816)

TP: true positive; FP: false positive; TN: true negative; FN: false negative; NPV: negative predictive value; PPV: positive predictive value.

## Data Availability

The data used to support the findings of this study are included within the article.
